# Methods for estimating the burden of antimicrobial resistance: a systematic literature review protocol

**DOI:** 10.1186/s13643-016-0364-8

**Published:** 2016-11-08

**Authors:** Nichola R. Naylor, Sachin Silva, Kavian Kulasabanathan, Rifat Atun, Nina Zhu, Gwenan M. Knight, Julie Robotham

**Affiliations:** 1 National Institute for Health Research Health Protection Research Unit in Healthcare Associated Infection and Antimicrobial Resistance at Imperial College, Hammersmith Campus, London, W12 0NN UK; 2 Imperial College London, Sir Alexander Fleming Building, South Kensington Campus, London, UK; 3 Harvard University, 665 Huntington Avenue, Boston, MA 02115 USA; 4 Modelling and Economics Unit, Public Health England, 61 Colindale Avenue, London, NW9 5EQ UK

**Keywords:** Antimicrobial resistance, Burden, Methods, Systematic review

## Abstract

**Background:**

Estimates of the burden of antimicrobial resistance (AMR) are needed to ascertain AMR impact, to evaluate interventions, and to allocate resources efficiently. Recent studies have estimated health, cost, and economic burden relating to AMR, with outcomes of interest ranging from drug-bug resistance impact on mortality in a hospital setting to total economic impact of AMR on the global economy. However, recent collation of this information has been largely informal, with no formal quality assessment of the current evidence base (e.g. with predefined checklists). This review therefore aims to establish what perspectives and resulting methodologies have been used in establishing the burden of AMR, whilst also ascertaining the quality of these studies.

**Methods:**

The literature review will identify relevant literature using a systematic review methodology. MEDLINE, EMBASE, Scopus and EconLit will be searched utilising a predefined search string. Grey literature will be identified by searching within a predefined list of organisational websites. Independent screening of retrievals will be performed in a two-stage process (abstracts and full texts), utilising a pre-defined inclusion and exclusion criteria. Data will be extracted into a data extraction table and descriptive examination will be performed. Study quality will be assessed using the Newcastle-Ottawa scales and the Philips checklists where appropriate. A narrative synthesis of the results will be presented.

**Discussion:**

This review will provide an overview of previous health, cost and economic definitions of burden and the resultant impact of these different definitions on the burden of AMR estimated. The review will also explore the methods that have been used to calculate this burden and discuss resulting study quality. This review can therefore act as a guide to methods for future research in this area.

**Systematic review registration:**

PROSPERO CRD42016037510

**Electronic supplementary material:**

The online version of this article (doi:10.1186/s13643-016-0364-8) contains supplementary material, which is available to authorized users.

## Background

Antimicrobial resistance (AMR) can be defined as the phenomenon in which microorganisms persist in the presence of antimicrobials, which are commonly used to prevent and/or treat infectious disease. AMR is a cause for concern within the UK and globally, due to the current and great potential negative impact on population health [1, 2]. AMR-associated burden can be defined as AMR impact on health (mortality or morbidity), impact on healthcare and patient costs or impact on the economy (labour force impact, productivity impact or opportunity cost) depending on study perspective. The AMR review, chaired by Jim O’Neill, has recently published estimates of potential future AMR burden, for example stating global gross domestic product (GDP) loss over the next 40 years could be as great as $3 trillion [1]. These estimates have since been cited by policy makers and the media [3, 4], showing the demand for estimates quantifying the current and potential future problem AMR poses. Accurate estimates of disease-related burden are needed for policy makers to establish disease-related resource need and advocate for appropriate levels of funding and are critical inputs for any health economic evaluations of AMR interventions.

Recent descriptive review articles have discussed methods of burden estimation in the context of AMR, citing a few articles as examples of different methodologies [5–7]. However, since the 2012 rapid review update of a previous systematic review [8, 9], there has been no systematic review which looks into the estimation of burden associated with AMR. None of the aforementioned reviews formally quality assess study methodology, which is needed to highlight methodological issues in establishing the burden of AMR. The 2012 rapid review by Smith and Coast [8] concluded that the evidence base suggests the burden of AMR is relatively modest due to the narrow perspective taken by most studies, and that a wider societal perspective was needed to capture the true impact. However, with more recent work taking a wider perspective on AMR burden [1] and many more research articles being published in this area in recent years, a new assessment is required of the current estimates of both health and economic AMR burden.

The aims of this systematic review include the following: (i) to establish what perspectives and resulting methodologies have been used in establishing the burden of AMR, (ii) to see how this impacts on the burden estimates given and (iii) to assess the quality of these studies.

## Methods/design

### Research question

What perspectives and resulting methodologies have been used in establishing the burden of AMR?

### Study overview

**Fig. 1:**
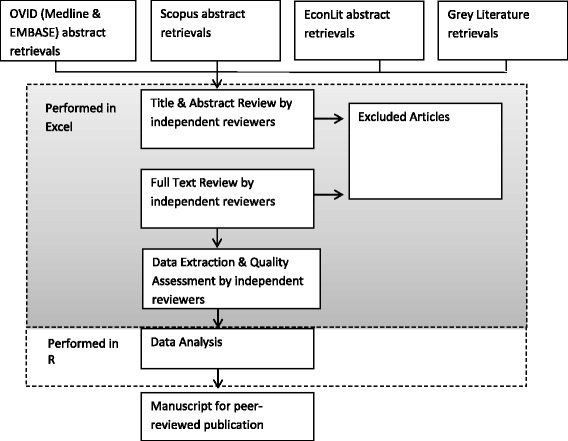
Overview of study methodology

Figure 1 depicts an overview of the study procedure.

### Study eligibility

Any studies that aim to quantify the burden of AMR within humans will be considered in this review, and this includes studies across any microbes, infections and country settings.

The modified PICO [10] inclusion and exclusion criteria to be applied at the review stages can be found in Table 1.

**Table 1:** Inclusion/exclusion criteria

Criteria	Inclusion	Exclusion
Population	Humans	Animals
	All ages	Plants
	All sexes	
	Infection with antimicrobial resistant organism	
Intervention	Not applicable	Not applicable
Comparator	Not applicable	Not applicable
Outcomes	Associated health burden, to include but not restricted to: Morbidity; for example excess length of stay in hospital Mortality	Health-related quality of life only
	Associated Healthcare cost burden, to include but not restricted to: Resource use Opportunity cost	Molecular biology only
	Economic burden, to include but not restricted to: Costs associated with loss of productivity and reduced labour force Work-loss hours per/case episode	Epidemiology only
	Secondary burden from not being able to use antibiotics in ways previously or currently used in healthcare, to include but not restricted to: Reduced surgery Reduced use in chemotherapy and similar therapies	Outcomes associated with the evaluation of an intervention such as clinical cure rate only
Study design	Case–control studies	Editorials
	Cohort studies	Letters
	Cross–sectional studies	Case series report
	Longitudinal studies	Conference abstracts/reports
	Randomised controlled trials	Evaluations of treatments/interventions
	Modelling studies	Reviews
	Economic evaluations	
Other	English language	

### Search strategy

The methods used in this systematic review are in line with the PRISMA guidelines [11]. In line with previous published protocols [12], a completed copy of the PRISMA-P checklist has been completed (see Additional file 1).

The search period will be restricted from 2013 onwards; this date was chosen to avoid retrieval duplication with Smith and Coast [8]. Ovid “Medline and EMBASE”, Scopus and EconLit will be searched, along with grey literature from predetermined agency websites. The following agency websites were defined after consulting a group of AMR researchers. Their content will be searched for reports and articles relating to the population of interest:

- Public Health England
- Public Health Wales
- Health Protection Scotland
- NHS Health Scotland
- Department of Health (UK)
- Health Protection Agency
- National Institute for Health and Care Excellence
- Centers for Disease Control and Prevention
- World Health Organisation
- European Commission for Public Health
- Review on Antimicrobial Resistance

It has been previously stated that many papers do not mention AMR generally, but rather specific microbes [8]. In an attempt to tackle this, an additional 13 clinically relevant bacteria will be highlighted in the search. These can be identified in the stated search string below (note that this is in the format for Scopus, the same terms are to be reformatted for OVID and EconLit searches):

((TITLE ((excess OR associated OR attributable) W/2 (burden OR morbidity OR mortality OR cost*)) OR ABS ((excess OR associated OR attributable) W/2 (burden OR morbidity OR mortality OR cost*))) OR (TITLE ((economic OR clinical OR global) W/2 (impact OR outcome* OR burden OR cost*)) OR ABS ((economic OR clinical OR global) W/2 (impact OR outcome* OR burden OR cost*)))) AND ((ALL ((“antibiotic” OR “antimicrobial” OR “multidrug” OR “microbial-drug”) PRE/1 resistan*)) OR ((TITLE (enterococc* OR escherichia OR streptococc* OR staphylococc* OR klebsiealla OR pseudomonas OR neisseria OR chlamydia OR clostridi* OR mycobacteri* OR “gram-positive” OR “gram-negative”) OR ABS (enterococc* OR escherichia OR streptococc* OR staphylococc* OR klebsiealla OR pseudomonas OR neisseria OR chlamydia OR clostridia* OR mycobacteri* OR “gram-positive” OR “gram-negative”)) AND ((TITLE (susceptib* OR nonsusceptib* OR resistan*) OR ABS (susceptib* OR nonsusceptib* OR resistan*)) OR (ALL ((“antibiotic” OR “antimicrobial” OR “multidrug” OR “microbial-drug”) PRE/1 resistan*)))))

The lead reviewer (NN) will review all abstracts and full texts. Independent reviewers will perform a parallel review of the abstracts and full texts, with each of these reviewers being assigned a percentage of the total retrieval items. Any discrepancies will be discussed and re-examined until agreement is reached.

### Quality assessment

Risk of bias in individual studies will be assessed using the Newcastle-Ottowa scales for cohort and case control studies [13], whilst the Philips checklist will be used for economic models [14]. These tools were chosen as the focus of this review is on study methodology rather than reporting standards.

Risk of bias across studies will be assessed in two groups; studies looking at health burden and studies looking at all other burden, and will simply be assessed based on the sign and significance of the outcome. This is due to the expected heterogeneity in studies (outcome, infection, resistance).

### Data collection and analysis

Data will be collected by the lead reviewer (NN). Data will be inputted into a standardised data extraction table (Excel) and independently checked to ensure quality.

The following information will be extracted: study identifiers, study characteristics (perspective, country setting), population characteristics, data setting (hospital or community), study methodology, outcome of interest (mortality, length of stay, cost), results (e.g. resistance has a significant impact on the outcome of interest), stated limitations and information used for risk of bias assessment (informed by the cited checklists).

A descriptive synthesis of the study information and risk of bias structured around the perspectives (health, health system and economic burden) and related methods used will be provided. This will include a results table containing individual level study data, and summary graphical representation of study characteristics such as scatter plots of estimates for excess mortality and monetary cost. We anticipate limited scope for a meta-analysis given the assumed heterogeneous nature of identified outcomes, studies included may differ across perspective, infection site, infection type/causative organism, bug-drug combinations and sub-populations. However, if there are suitable data for one drug-bug combination in similar populations, then forest plots will be constructed utilising hazard ratio as the comparative outcome [15].

The format of this write-up will be a manuscript which will be submitted for publication in a peer-reviewed journal, it will also contribute to the lead reviewer’s (NN) PhD project as part of the National Institute for Health Research Health Protection Research Unit (NIHR HPRU) in Healthcare Associated Infections and Antimicrobial Resistance.

## Discussion

Recent estimates for the burden suggest that AMR that it is a significant economic burden to the global economy [1], whilst previous reviews have suggested that perhaps study outcome and methodology impacts whether AMR is found to have a significant burden or not [8]. Yet, there has been no literature review which formally looks to assess the quality of such studies.

Originally, the leading author ran a similar search strategy independently; however, after discussion with co-authors, it was realised that given the nature of previous reviews, and the lack of quality assessment of previous literature, the original study design did not adequately answer the research question or fill the current research gap. Therefore, the original study was halted (results not published in peer-review) and the study protocol was revised into the protocol written here.

This review will provide an overview of previous health, cost and economic definitions of burden in the context of AMR. The review will also explore the methods that have been used to calculate this burden and discuss resulting study quality. This review can therefore act as a guide to methods for future research.

## Additional file

Additional file 1: PRISMA-P (Preferred Reporting Items for Systematic review and Meta-Analysis Protocols) 2015 checklist: recommended items to address in a systematic review protocol. (DOC 81 kb)

## Abbreviations

AMR: Antimicrobial resistance
GDP: Gross domestic product
PICO: Population, Intervention, Comparison, Outcome
UK: United Kingdom
